# Implications of Public Understanding of COVID-19 in Saudi Arabia for Fostering Effective Communication Through Awareness Framework

**DOI:** 10.3389/fpubh.2020.00494

**Published:** 2020-09-18

**Authors:** Fahad Alanezi, Anan Aljahdali, Seham Alyousef, Hebah Alrashed, Wyam Alshaikh, Hayat Mushcab, Turki Alanzi

**Affiliations:** ^1^Community College, Imam Abdulrahman Bin Faisal University, Damam, Saudi Arabia; ^2^Biological Science Department, Faculty of Science, University of Jeddah, Jeddah, Saudi Arabia; ^3^Department of Community and Psychiatric Mental Health Nurse, Nursing College, King Saud University, Riyadh, Saudi Arabia; ^4^King Saud University, Riyadh, Saudi Arabia; ^5^King Faisal Specialist Hospital & Research Centre, Riyadh, Saudi Arabia; ^6^Johns Hopkins Aramco Healthcare (JHAH), Dhahran, Saudi Arabia; ^7^Department of Health Information Management and Technology, College of Public Health, Imam Abdulrahman Bin Faisal University, Damam, Saudi Arabia

**Keywords:** COVID-19, awareness framework, infectious disease, pandemics, public awareness

## Abstract

**Background:** Participation of the public is an important and most effective approach for controlling the spread of novel coronavirus. However, considering its novel nature, it is important to create awareness among the public to be able to take timely preventive measures. On the contrary, misinformation and myths from online communities result in severe damages in mitigation of this novel disease.

**Objective:** Focusing on these aspects, this manuscript reviews public awareness about COVID-19, myths surrounding it, its symptoms, treatment, transmission, importance of information sources, types of information to be considered in awareness campaigns, promotional channels, and their implications in Saudi Arabia.

**Methods:** An online questionnaire-based survey was used for collecting data related to five major aspects related to COVID-19 and awareness creation process. The survey was accessed by 1,881 people, out of whom 741 people participated in the survey. However, 150 dropouts left the survey in between, as a result of which a final sample of 591 was achieved, indicating the response rate of 39.3% and a completion rate of 79.76%.

**Results:** Awareness levels of the participants related to COVID-19, its means of transmission, preventive measures, symptoms, and treatment were identified to be moderate to high (60–80%). However, reliance on a few myths and violation of certain preventive measures were identified with majority of the participants (more than 60%). The Ministry of Health was identified to be the most reliable source of information followed by family and friends. Moreover, 15 types of information were identified to be highly relevant and important, which need to be effectively disseminated among the public through effective communication channels.

**Conclusions:** Lack of awareness can result in serious outcomes in relation to COVID-19. Effective awareness campaigns including relevant information from reliable sources can improve the knowledge of people, and they must be effective in developing positive attitudes among the public toward adopting preventive measures.

## Introduction

Creating public awareness about infectious diseases which are caused by new pathogens is one of the effective approaches for controlling the spread of diseases such as COVID-19. As the information about the disease, its symptoms, precautionary methods, diagnosis, and treatment may vary with other infectious diseases and it may take considerable amount of time, it is important for timely updates about the pandemic and the preventive care to be disseminated among the public in order to contain the transmission of infection.

Lack of public awareness about COVID-19 was observed in different places in the initial days of the pandemic, and people roamed freely without following precautionary methods such as social distancing, and wearing masks (1–3). While the nature of the pandemic changes, it is important that the information and advice remain constant. Therefore, it is very important that accurate and reliable information must be disseminated to the public through verified sources, and spread of any misinformation must be effectively contained to prevent any loss. Therefore, various reliable sources including the World Health Organization (WHO) and United Nations sister organizations, along with governments of various countries, have been providing regular updates and the necessary information to prevent COVID-19 through various channels (4–6).

Another important factor of creating awareness is to prevent the spread of myths and misinformation. It is evident that perceptions and myths such as drinking raw alcohol can cure COVID-19 by people in Iran (7), that 5G towers are the cause for COVID-19 by people in the UK (8), and eating garlic or mint can cure COVID-19, as well as many others (9), can lead to serious damage and may increase the chances of contamination. A recent study has identified that there is a positive correlation between the increase in the number of COVID-19 cases and the relative search volumes of terms related to COVID-19 (10).

In addition, public awareness about COVID-19 varied across sub-regions in different countries, and the immediate need for strengthening the publicity regarding COVID-19 by the governments was identified. However, the concerns about the transmission and the number of infected persons is growing at alarming rates in the past few months compared to other diseases like SARS, MERS-CoV, and Influenza. A recent review (11) of various studies in China and other countries related to COVID-19 has indicated that the reproductive rate (it is an indication of the transmissibility of a virus, representing the average number of new infections generated by an infectious person in a totally naive population) of COVID-19 is very high compared to other infectious diseases. In addition, children and old-aged people are identified to be at high risk of contamination with the novel coronavirus if necessary precautionary methods were not taken. Studies have identified that infection was mainly identified in family clusters and workplaces (12), reflecting the transmission by direct or close contact in the environment of those with infection.

On the other hand, the governments are adopting various approaches (12) such as containment and mitigation activities to delay the major surges in number of patients and level the demand for healthcare resources such as hospital beds, testing kits, medicines, and other medical equipment and also to protect the most vulnerable from infection, including elderly people and those with health complexities or other critical diseases (13, 14). Considering these approaches by the governments, it is important that people are provided with accurate and timely information in relation to these approaches. Focusing on the aspect of public awareness, this paper investigates the level of public awareness in Saudi Arabia and analyzes the types of information to be communicated from the reliable sources and its implications on the public by proposing a conceptual framework.

## Methods

The purpose of this study was to investigate the level of public awareness about COVID-19 in Saudi Arabia and the importance of information sources, information types, and communication/promotional channels for creating awareness among the people in Saudi Arabia. As an approach for achieving this objective, an online questionnaire-based survey was adopted.

The questionnaire was designed with various aspects related to COVID-19 and level of awareness. It included various sections, including questions related to general awareness of COVID-19 (four items), its symptoms (six items), transmission (three items), preventive care (10 items), treatment options (two items), myths (eight items), types of information (15 items), communication/promotional channels (nine items), and sources of information (five items). Multiple-choice answers and five-point Likert scale ratings (15) were used by the participants to answer the questions.

The questionnaire was initially designed in English and was then translated to Arabic by two professional Arabic translators. The Arabic version of the questionnaire was designed using QuestionPro application. A pilot study was conducted with 12 randomly selected people for evaluating the questionnaire. Based on the feedback from the pilot study participants, few changes were made in relation to the questions' formulation and grammatical errors in Arabic. In addition, Cronbach's alpha for all items in the questionnaire was identified to be >0.88, revealing good consistency and reliability.

### Recruitment

The general public living in Saudi Arabia were recruited for the survey using the survey link generated using QuestionPro application. The survey link was initially forwarded to the general public by posting the link on community groups and other platforms on social media platforms. Moreover, the survey was conducted for a period of 4 weeks from 23 March to 19 April 2020.

### Sampling

Considering the purpose and objective of the study, which was to collect the data from the general population of Saudi Arabia, the participants were randomly selected. However, the targeted sample population was composed of adults aged 18 years or above. As an approach to reach maximum samples in a short time, snowball sampling technique (16) was adopted, in which a request is made while forwarding the survey link, whereby participants were requested to forward the message to their friends and colleagues. Accordingly, the survey link was initially forwarded to 439 people through various modes. As a result of using snowball sampling technique, the link was accessed by 1,881 people, out of which 741 people participated in the survey. However, 150 dropouts were identified who left the survey in between; as a result a final sample of 591 was achieved, indicating a response rate of 39.3% and a completion rate of 79.76%. In addition, the average time taken by the participants to complete the survey was 7 min.

### Analytical Process

The survey was developed using QuestionPro application and conducted for a period of 4 weeks. The data were analyzed and discussed using four themes, which included sources of information, types of information, communication/promotional channels, and implications of good public awareness. Relative frequencies for each item under these themes are used for analyzing the data, which are presented in the following section.

## Results

The final sample achieved in this study was 591. The demographic information of the participants is presented in Table 1. Among the total participants, 65.31% were male and 34.69% were female. Considering the age groups, 59.05% were aged between 25 and 34 years followed by 16.07% between 45 and 54 years, 13.36% between 35 and 44 years, 9.47% between 18 and 24 years, and only 12 participants aged more than 54 years. Focusing on the education levels of the participants, 57.39% have bachelor's degrees, followed by 14.25% who have master's degrees, 12.89% have Diploma, 11.13% have Ph.D., and 21 participants have secondary education.

**Table 1:** Frequency distribution of demographic variables.

**Variables**	***n***
**Gender**	
Male	386
Female	205
**Age**	
18–24	56
25–34	349
35–44	79
45–54	95
>54	12
**Education**	
Secondary education	21
Diploma	77
Bachelor's degree	341
Master's degree	85
Ph.D.	67
**Profession**	
Government employee	123
Private sector employee	117
Business	159
Student	63
Retired	43
Unemployed	86
**Region**	
Mecca	109
Medina	198
Riyadh	169
Other regions	115
**Is your education background related to healthcare practices/healthcare management?**	
Yes	84
No	507
**Are you working in any healthcare related organization?**	
Yes	92
No	499

Focusing on the professions of the participants, a diverse scenario can be observed with 20.81% government employees, 19.79% private sector employees, 26.90% business professionals, 10.65% students, 14.45% unemployed, and 7.27% retired individuals. Majority of the participants belonged to three regions: 33.52% from Medina, 28.73% from Riyadh, 18.33% from Mecca, and 19.42% belonged to other regions of Saudi Arabia. It is important to note that 85.78% of the participants' educational background (degree education) was not related to healthcare and 84.44% of the participants were not working in healthcare-related organizations. Working in healthcare organizations or having a qualification related to healthcare may increase the possibility that the participants were more aware of the infectious diseases/healthcare aspects compared to other participants.

Focusing on the general awareness of COVID-19, majority of the participants, 86.31%, identified incubation period (the time between catching the virus and beginning to have symptoms of the disease) to be ranging from 5 to 14 days, while 12.68% of the participants stated they do not know, and six participants stated 21 days. In addition, 83.6% of participants were aware that COVID-19 is a disease caused by novel coronavirus, and 91.5% of participants believed it was identified in Wuhan region, China. While 36.25% of participants believed that the source of the novel coronavirus is “bats,” 14.35% believed the source is “Chinese man;” 49.4% stated that the source is not yet identified. In addition, there are various myths being circulated online, and the participants' awareness levels in relation to these myths are presented in Table 2. The findings reflected that 18% of the participants believed various myths circulating online, which are not officially confirmed or declared by the governments or healthcare organizations.

**Table 2:** Relating frequencies (%) related to various myths.

**Myths**	**True (%)**	**False (%)**
New coronavirus cannot be transmitted in hot and humid climates.	23.89%	76.11%
Cold weather can kill new coronavirus.	21.20%	78.80%
The new coronavirus can be transmitted through mosquito bites.	26.34%	73.66%
Spraying alcohol or chlorine all over your body kills the new coronavirus.	31.52%	68.48%
Hand-dryers are effective in killing new coronavirus.	33.61%	66.39%
Vaccines against pneumonia protect you against the new coronavirus.	22.55%	77.45%
Regularly rinsing your nose with saline can help in preventing infection with the new coronavirus.	18.96%	81.04%
Eating garlic can help in preventing infection with the new coronavirus.	32.45%	67.55%

Public awareness about COVID-19 symptoms is presented in Table 3, which has revealed that majority of the participants (84.26%) identified fever, dry cough, and breathing difficulties as the general symptoms of COVID-19, and prolonged illness or symptoms in severe cases as identified by 86.63% of the participants may include pneumonia, acute respiratory syndrome, and organ failure.

**Table 3:** Relative frequencies (%) related to COVID-19 symptoms.

**Symptoms**	***N***	**Relative frequency (%)**
**General symptoms**		
Fever	38	6.43%
Dry cough	24	4.06%
Breathing difficulties	31	5.25%
All of the above	498	84.26%
**Symptoms in severe cases**		
Pneumonia	39	6.60%
Acute respiratory syndrome	13	2.20%
Organ failure	27	4.57%
All of the above	512	86.63%

Similarly, public awareness about the transmission risks is presented in Table 4. Majority of the participants (76.48%) identified different possibilities of transmission by not adopting social distancing measures.

**Table 4:** Relative frequencies (%) related to COVID-19 transmission.

**Modes**	***N***	**Relative frequency (%)**
When a person sneezes or coughs, droplets spread in the air or fall on the ground and nearby surfaces.	74	12.52%
If another person is nearby and inhales the droplets or touches these surfaces and further touches his face, eyes or mouth, he or she can get an infection.	29	4.91%
If the distance is <1 m from the infected person.	36	6.09%
All of the above	452	76.48%

In relation to the possibility of cure and treatment, it was acknowledged by 83.65% of the participants that most of the affected persons may recover on their own, and only a small proportion of patients who have severe pre-medical conditions, are old-aged, and are children may need intensive care. It is interesting to note that 74.79% of the participants were aware that people with chronic acute respiratory disease can be severely affected if they are infected with novel coronavirus. In addition, 69.43% of the participants were aware that there is no treatment available for COVID-19, but about 30% believed that there is a treatment available, which may be an issue of concern, as they may not seriously adopt preventive measures. Focusing on the public awareness of preventive measures, Table 5 indicated good awareness levels, as 70–99% of participants acknowledged different preventive measures.

**Table 5:** Relating frequencies (%) related to COVID-19 preventive measures.

**Actions**	***N***	**Relative frequency (%)**
Wash your hands with soap and water for at least 20 s.	436	73.77%
Use an alcohol-based hand sanitizer.	459	77.66%
Cover your mouth and nose with a tissue while sneezing.	563	95.26%
Always wear a protective N-95 mask.	521	88.16%
Maintain social distancing (at least 1 m distance from others).	542	91.71%
Avoid unprotected/close contact with anyone developing cold/flu like symptoms.	536	90.69%
Avoid unprotected/direct contact with live animals and surfaces in contact with animals, when visiting a market.	499	84.43%
Cook your food, especially meat, thoroughly.	478	80.88%
Self-quarantine at home for at least 14 days, if you feel any symptoms such as fever, cold, and cough.	486	82.23%
Seek medical care, if these symptoms prolong.	581	98.31%

However, only 78.85% of the participants stated that they always followed precautionary methods, while 12.96% stated they followed sometimes, and 8.19% stated that they did not follow any precautionary methods. However, 97.6% of the participants believed that quarantine and staying at home is an effective approach toward preventing the spread of novel coronavirus. In addition, only 32.29% of the participants stated that they did not leave home during lockdown/curfew, while 54.2% stated they left home as it was necessary, and 13.51% stated that they left home without any reason. Accordingly, 30.64% stated they left home once (1 day) a week, 12.03% 2 days per week, 6.75% 3 days per week, 3.95% 4 days per week, 1.98% 5 days per week, and 2.80% 6 days per week; 32.62% stated they did not leave the house.

In relation to the reliable sources of information, participants were asked about various sources which they would prefer, and the results are presented in Table 6, which indicates that majority of the participants relied on the Ministry of health, friends, and family.

**Table 6:** COVID-19 information sources.

**Sources**	***N***	**Relative frequency (%)**
Ministry of Health	386	65.31%
Friends and relatives	412	69.71%
Recognized bodies such as World Health Organization	108	18.27%
Research organizations	56	9.48%
Experts	197	33.33%

In addition, the participants were asked to rate the importance and effectiveness of various types of information which need to be promoted, and the findings are presented in Table 7. Although all types of information were important, few types such as access to care, helpline and support, health insurance, and access to medicine were highly important.

**Table 7:** Types of information for COVID-19 awareness and management.

**Types**	**Strongly agree**	**Agree**	**Neutral**	**Disagree**	**Strongly disagree**
Diagnostics	69.30%	8.96%	4.32%	5.86%	11.56%
Symptoms	78.68%	11.31%	2.14%	6.38%	1.49%
Preventive care	65.89%	7.63%	5.97%	10.86%	9.65%
Treatment option	71.60%	9.82%	11.32%	2.11%	5.15%
Medication	78.25%	9.66%	8.32%	1.64%	2.13%
Lifestyles	52.13%	21.72%	5.31%	11.82%	9.02%
Access to care	81.67%	5.41%	7.64%	3.26%	2.02%
Access to medicine	79.48%	10.32%	5.65%	3.25%	1.30%
Helplines and support	85.45%	6.24%	3.16%	4.50%	0.65%
Associated risks	57.36%	21.89%	11.58%	5.15%	4.02%
Transmission information	69.30%	8.96%	4.32%	5.86%	11.56%
Myths and misinformation	78.68%	11.31%	2.14%	6.38%	1.49%
Government decisions and strategies	65.89%	7.63%	5.97%	10.86%	9.65%
Travel and business	71.60%	9.82%	11.32%	2.11%	5.15%
Health insurance	78.25%	9.66%	8.32%	1.64%	2.13%

Similarly, participants were asked to rate the importance and effectiveness of various channels/modes of communication, and the responses are presented in Table 8, which indicated online government portals and mobile [calls/SMS (short message service)] were identified to be important.

**Table 8:** Communication channels for creating awareness.

**Modes**	**Strongly agree**	**Agree**	**Neutral**	**Disagree**	**Strongly disagree**
Social media	55.45%	32.82%	5.65%	4.32%	1.76%
Other online platforms (government portals, press releases, etc.)	87.32%	6.41%	2.32%	3.18%	0.77%
Television	71.25%	12.96%	5.45%	6.21%	4.13%
Radio	63.89%	8.65%	4.95%	10.85%	11.66%
Mobiles	72.19%	12.98%	6.78%	4.32%	3.73%
Newspapers	45.21%	6.82%	13.98%	14.55%	19.44%
Community centers	52.98%	10.65%	9.87%	16.25%	10.25%
Non-government organizations	58.95%	13.52%	12.97%	6.85%	7.71%
Local campaigns	63.50%	17.98%	13.54%	3.40%	1.58%

## Discussion

The findings related to public awareness have revealed some important aspects related to the information known by the public and the implications especially in adopting preventive measures. In addition, the information flow, reliable sources, types of information, and modes of promotions can be assessed in the context of Saudi Arabian lifestyle. Firstly, focusing on the general awareness about COVID-19, participants exhibited good understanding about the disease, the pathogen causing the disease, its sources, and the incubation period. Though the source of COVID-19 is yet to be identified, there are a considerable number of participants who believed the source of the virus might be bats or transmitted through Chinese people.

In relation to the awareness about myths circulating online and the truth in them, most of the participants reflected good understanding of the myths, which were verified by the World Health Organization (9) and turned out to be false. However, in relation to few myths, there are a considerable number of participants (~30% of the participants) who believed them to be true, such as using alcohol, hand dryers, and eating garlic can kill the virus. These can have serious outcomes, as it is evident from the recent incidents such as drinking raw alcohol in Iran (7) and burning down 5G towers in the UK (8). Therefore, the spread of such myths must be targeted by effectively promoting awareness campaigns through various channels.

Focusing on the symptoms, participants reflected good understanding, as they stated fever, dry cough, and breathing difficulties as general symptoms which were identified by various reliable organizations (17–19). One of the important aspects of COVID-19 awareness is related to the various means of transmission from an infected person. In relation to these factors, most of the participants reflected good understanding, as they identified that the main cause of virus spread is through the droplets released by an infected person through sneezing or coughing, which can rest on different places for a considerable amount of time. However, one of the concerns is that about 24% of the participants were not aware of these factors. Unlike other infections, the importance of awareness and preventive measures is very important in containing the spread of COVID-19, as there is a high risk of contamination from a single person which can easily lead to the infections across the community or region if proper precautionary methods are not implemented (20, 21).

Focusing on awareness of preventive measures, participants exhibited good understanding, especially in relation to social distancing, covering mouth and nose while coughing or sneezing, avoiding close contact with symptomatic (flu, cough) persons, and seeking medical help in case the symptoms prolong after incubation period during quarantine. However, other preventive measures such as washing hands regularly and using hand sanitizers were only recognized by ~75% of the participants. These two approaches are among the important measures which need to be considered on a daily basis to prevent being infected and contain the spread of the virus (22).

In relation to the reliable sources of information about COVID-19, majority of the participants relied more on the Saudi Ministry of Health, friends, and relatives than on the recognized bodies such as WHO and healthcare experts. It is important that the public should rely on reliable sources of information, as unreliable sources increase the chances of contamination and other challenges related to healthcare and social challenges as a result of vast misinformation available on various channels (23, 24).

In relation to the types of information to be considered during COVID-19 outbreak, there has been no consensus among the organizations. However, information related to preventive measures, symptoms, and self-care were the most promoted (10, 25–27); there is a need for considering the additional information in order to prevent the spread of mis-information, enable people to manage their activities during lockdown/quarantine, and manage their lifestyles and other aspects such as finance, basic needs, and other necessary aspects. Therefore, various types of information were reviewed, and 15 different types of information (presented in Table 7) were perceived to be highly important by most of the participants.

Focusing on the channels/modes of promotion, it is essential to consider that information must be disseminated to a large section of the population within a short time, and it is also essential that regular updates can be easily accessed by the public. Social media and mobile phones (SMS/calls) can be effective in reaching a large section of the population in a short time. Therefore, approaches such as passing messages and information about COVID-19 before connecting a call on mobiles by the mobile services companies and daily SMS and mobile applications launched by the government to create awareness and track diseases and vulnerability of the users having an infection are proving to be effective in different regions (28–32). However, majority of the participants preferred online government portals and press releases compared to social media platforms. In addition, mobiles and television were considered by the participants to be effective platforms for creating awareness. It is interesting to note that newspapers were least preferred compared to other channels, as the risk of contamination may be high.

By effectively creating public awareness, the spread of COVID-19 can be minimized, and the risk of infections, death, and losses can be prevented. It can also result in effective health outcomes, improve quality of life during lockdowns, survival, and proper planning of work, business and finances, etc.

**Figure 1:**
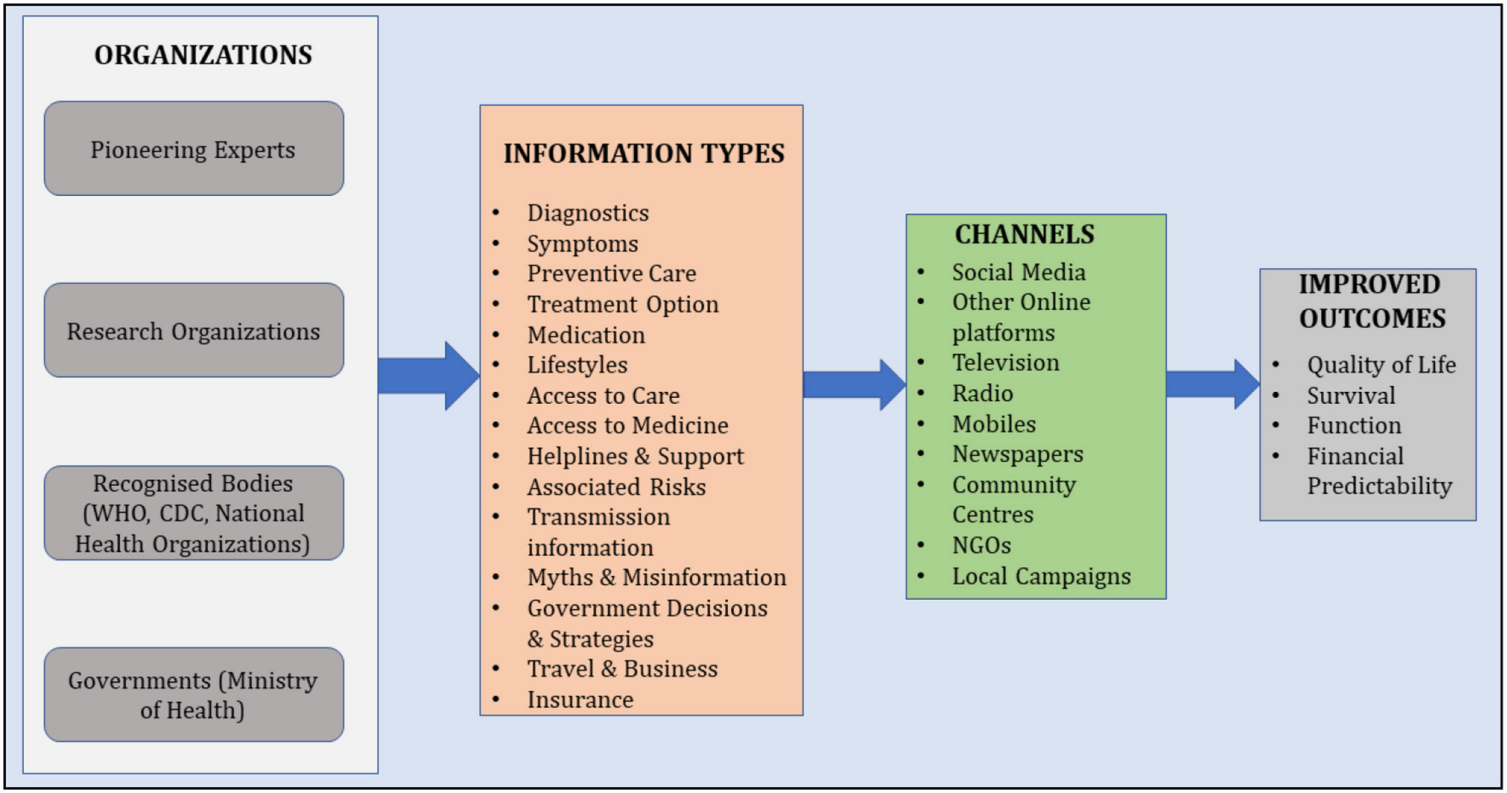
Framework for public awareness during COVID-19 outbreak.

Based on these findings, a framework (Figure 1) for creating public awareness with components including information sources, types of information, communication channels, and the outcomes is formulated especially considering Saudi Arabian lifestyle. This framework can also be used as conceptual framework for future studies focusing on evaluating public awareness related to pandemics/infectious diseases.

### Limitations

There are a few limitations in this study. The first is the methodological approach based on survey questionnaire for collecting and analyzing the public awareness data related to COVID-19; a mixed method approach such as observations and interviews could have gathered more qualitative and behavioral data which can be used to analyze the public reactions and lifestyle changes in relation to COVID-19 outbreak. In addition, the survey was conducted over a period of 4 weeks, which could have been increased to achieve a large sample population and response rates. A major limitation of this study is the online questionnaire due to the lockdown situation that reduced the reachability to boarder communities with good sample pool.

### Implications

Various implications can be drawn from the study. Firstly, this study contributes to the literature by providing the relationship between awareness and self-care practices adopted by the public considering the COVID-19 outbreak, reflecting the people's attitudes toward the pandemic and preventive measures. The findings from the survey can prove to be a valuable source of information for the government, based on which it can update its awareness creation strategies and also tract peoples' attitudes toward the pandemic. In addition, the proposed framework can also be used as a conceptual framework in other research studies focusing on public awareness about pandemic/infectious diseases.

## Conclusion

This study analyzed the public awareness about COVID-19, its precautionary measures, and its implications on the lifestyles of the people in Saudi Arabia. An online survey was conducted, considering the prevailing situation of lockdown to reach maximum participants. A total of 591 respondents participated in this survey. Overall, the findings revealed that public awareness about COVID-19 in Saudi Arabia varied between moderate to high, and its implications reflected that a few measures were not adopted by the public, such as staying at home, which resulted in increased number of positive cases. Though they were aware of the precautionary measures of staying at home during lockdowns, most of the participants frequently went out of their homes, which might increase the risk of contamination. Therefore, it is very much essential that strict measures and an effective approach for creating awareness are to be adopted, to ensure the success of the lockdown strategy in order to limit the spread of COVID-19.

## Data Availability Statement

The original contributions presented in the study are included in the article/supplementary material, further inquiries can be directed to the corresponding author/s.

## Ethics Statement

The studies involving human participants were reviewed and approved by The Institutional Review Board of the Imam Abdulrahman Bin Faisal University. The patients/participants provided their written informed consent to participate in this study.

## Author Contributions

All authors listed have made a substantial, direct and intellectual contribution to the work, and approved it for publication.

## Conflict of Interest

The authors declare that the research was conducted in the absence of any commercial or financial relationships that could be construed as a potential conflict of interest.
